# Single Independent Autopolyploidization Events From Distinct Diploid Gene Pools and Residual Sexuality Support Range Expansion of Locally Adapted Tetraploid Genotypes in a South American Grass

**DOI:** 10.3389/fgene.2021.736088

**Published:** 2021-10-04

**Authors:** Piyal Karunarathne, Diego Hojsgaard

**Affiliations:** ^1^ Department of Systematics, Biodiversity and Evolution of Plants, Albrecht-von-Haller Institute for Plant Sciences, University of Goettingen, Goettingen, Germany; ^2^ Georg-August University School of Science, University of Goettingen, Goettingen, Germany; ^3^ Evolutionary Biology Center, Uppsala University, Uppsala, Sweden; ^4^ Taxonomy & Evolutionary Biology, Leibniz Institute of Plant Genetics and Crop Plant Research (IPK), Gatersleben, Germany

**Keywords:** apomixis, residual sexuality, *Paspalum intermedium*, polyploid establishment, contact zone

## Abstract

Polyploidy plays a major role in plant evolution. The establishment of new polyploids is often a consequence of a single or few successful polyploidization events occurring within a species’ evolutionary trajectory. New polyploid lineages can play different roles in plant diversification and go through several evolutionary stages influenced by biotic and abiotic constraints and characterized by extensive genetic changes. The study of such changes has been crucial for understanding polyploid evolution. Here, we use the multiploid-species *Paspalum intermedium* to study population-level genetic and morphological variation and ecological differentiation in polyploids. Using flow cytometry, amplified fragment length polymorphism (AFLP) genetic markers, environmental variables, and morphological data, we assessed variations in ploidy, reproductive modes, and the genetic composition in 35 natural populations of *P. intermedium* along a latitudinal gradient in South America. Our analyses show that apomictic auto-tetraploids are of multiple independent origin. While overall genetic variation was higher in diploids, both diploids and tetraploids showed significant variation within and among populations. The spatial distribution of genetic variation provides evidence for a primary origin of the contact zone between diploids and tetraploids and further supports the hypothesis of geographic displacement between cytotypes. In addition, a strong link between the ecological differentiation of cytotypes and spatial distribution of genetic variation was observed. Overall, the results indicate that polyploidization in *P. intermedium* is a recurrent phenomenon associated to a shift in reproductive mode and that multiple polyploid lineages from genetically divergent diploids contributed to the successful establishment of local polyploid populations and dispersal into new environments.

## Introduction

Polyploidization in plants is a recurring and pivotal evolutionary phenomenon that brings both short- and long-term benefits for plant diversification (Werth et al., 1985; Soltis et al., 2010; Symonds et al., 2010). Comparative genomic studies show that approximately 15% of plant speciation events resulted from polyploidy (Wood et al., 2009) and that polyploidy is substantially associated to higher plant diversity (Symonds et al., 2010; Jiao et al., 2011). A crucial step of polyploidization, the formation of unreduced gametes, occurs at a noticeable rate of ∼0.5% per gamete (Ramsey and Schemske, 1998; Wood et al., 2009). Yet, the occurrence of new polyploids in natural populations is unequivocally lower than expected from such values (Suda and Herben, 2013), implying that polyploidization events are not always inherently beneficial. Polyploidization can either act as an instantaneous mechanism for divergence and speciation because of reproductive isolation (see Soltis et al., 2009) or as a step toward extinction due to competitive exclusion by the majority cytotype [i.e., minority cytotype disadvantage (Levin, 1975; Parisod et al., 2010)]. Therefore, mechanisms that help newly arisen polyploids to overcome competition, survive, and establish themselves devoid of reproductive isolation are key to the success of polyploids.

Apomixis (asexual reproduction *via* seeds) is a reproductive strategy almost exclusively coupled with polyploidy. Since apomictic plants skip meiosis and fertilization and form clonal euploid seed embryos, apomixis can be particularly beneficial during the establishment of new polyploids by shielding triploids from the negative effects of unbalanced chromosomal segregation, low density, and parental introgression, thus aiding new polyploids to overcome reproductive exclusion (Hojsgaard, 2018). Thus, apomixis provides reproductive assurance to polyploids (Levin, 1975) and enhances a plant’s ability to colonize new habitats reinforcing founder events (Baker, 1955). Since meiosis is bypassed during offspring formation, apomixis maintains heterozygosity levels and may even counteract genetic drift during plant dispersals (Paun et al., 2006; Cosendai et al., 2013). Furthermore, additional genetic variation among clonal individuals in apomictic populations comes from mutation accumulation and residual sexuality (Hörandl and Paun, 2007; Hojsgaard and Hörandl, 2015). Such variations provide polyploids with the genetic novelty needed to adapt to environments and expand their range. Many apomictic polyploid complexes display a pattern called geographical parthenogenesis, whereby sexual cytotypes occupy restricted geographical areas while apomicts are more widespread, often into higher latitudes (Brochmann et al., 2004; Suda et al., 2004; Hörandl, 2006).

The observed ecological amplitude or tolerance to wider, sometimes extreme, environmental conditions in apomictic polyploids compared to their sexual parents is usually explained through two main hypotheses: The General Purpose Genotype hypothesis (GPG) (Baker, 1967; Lynch, 1984) explains that one fit genotype with higher tolerance to a broader ecological setting may colonize different habitats, while the Frozen Niche Variation (FNV) hypothesis assumes that single genotypes can freeze a portion of the genetic variation of the sexual progenitors and therefore adapt to restricted ecological setups, thus efficiently partitioning underutilized resources by the ancestors (Vrijenhoek, 1984, 1994). Although these two concepts are mutually exclusive, recent studies suggest that mechanisms of inter-clonal selection acting upon natural asexual populations to acquire their ecological breadth and clonal divergence support both hypotheses (Vrijenhoek and Parker, 2009).

Cytotype coexistence implies the possibility of spontaneous exchanges of genetic information between ploidy levels. Inter-cytotype gene flow will result in patterns of genetic admixture, like observed in different polyploid apomictic complexes (Petit et al., 1999; Paun et al., 2006; Zozomová-Lihová et al., 2015). In general, gene flow is mostly unidirectional as it mainly takes place from diploids to polyploids, but cases of genetic introgression from polyploids to diploids have also been observed (Bretagnolle and Thompson, 1996; Van Dijk and Bakx-Schotman, 1997; Ramsey and Schemske, 1998). Recurrent polyploidization events from genetically divergent diploids are common in either sexual (e.g., Soltis et al., 2004) or apomictic polyploids (e.g., Sharbel and Mitchell-Olds, 2001) and can create genetically distinct populations or introduce novel genetic material in an established population to be recombined and assorted among polyploid individuals during sexual reproduction.

Studies of extant genetic variation in polyploid populations compared to their putative diploid parents not only provide evidence for the dynamic formation and fate of polyploids in natural populations (Soltis and Soltis, 2000); they also shed light on their recent evolutionary history and mechanisms of genetic and ecological differentiation (Fox et al., 2020).

*Paspalum intermedium* Munro ex Morong is a grass species of the sub-family Panicoideae with two major cytotypes: self-sterile sexual diploids (2n = 2x = 20) and self-fertile apomictic autotetraploids (2n = 4x = 40) (Norrmann et al., 1989). Despite the species occurring in a wide range of ecological and climatic gradients in sub-tropical South America (Zuloaga et al., 2012), a recent study revealed a significant shift in niche-optima between diploids and tetraploids and a dynamic competition and displacement between cytotypes in an ecotone where populations are segregated in allopatry, sympatry, and parapatry (Karunarathne et al., 2018). Therefore, *P. intermedium* makes an ideal non-model plant system to examine extant levels of genetic variation and associated traits to better understand the formation and dynamics of polyploids, the main drivers of habitat adaptation, niche divergence, and cytotype coexistence and to get a glimpse of the recent evolutionary history of the complex.

In the present study, using flow cytometry, amplified fragment length polymorphism (AFLP) markers, and ecological data, we endeavor 1) to assess extant levels of genetic variability in *P. intermedium* populations; 2) to evaluate the differences in population structure between cytotypes with divergent reproductive modes; 3) to examine the genetic composition of sympatric, parapatric, and allopatric populations in the contact zone; and 4) to search for associations between genetic variability parameters and distribution patterns which may support alternative ecological hypothesis and population attributes of different cytotypes that may help to clarify the recent evolutionary history of the species.

## Materials and Methods

### Sampling and Cytotyping

**FIGURE 1:**
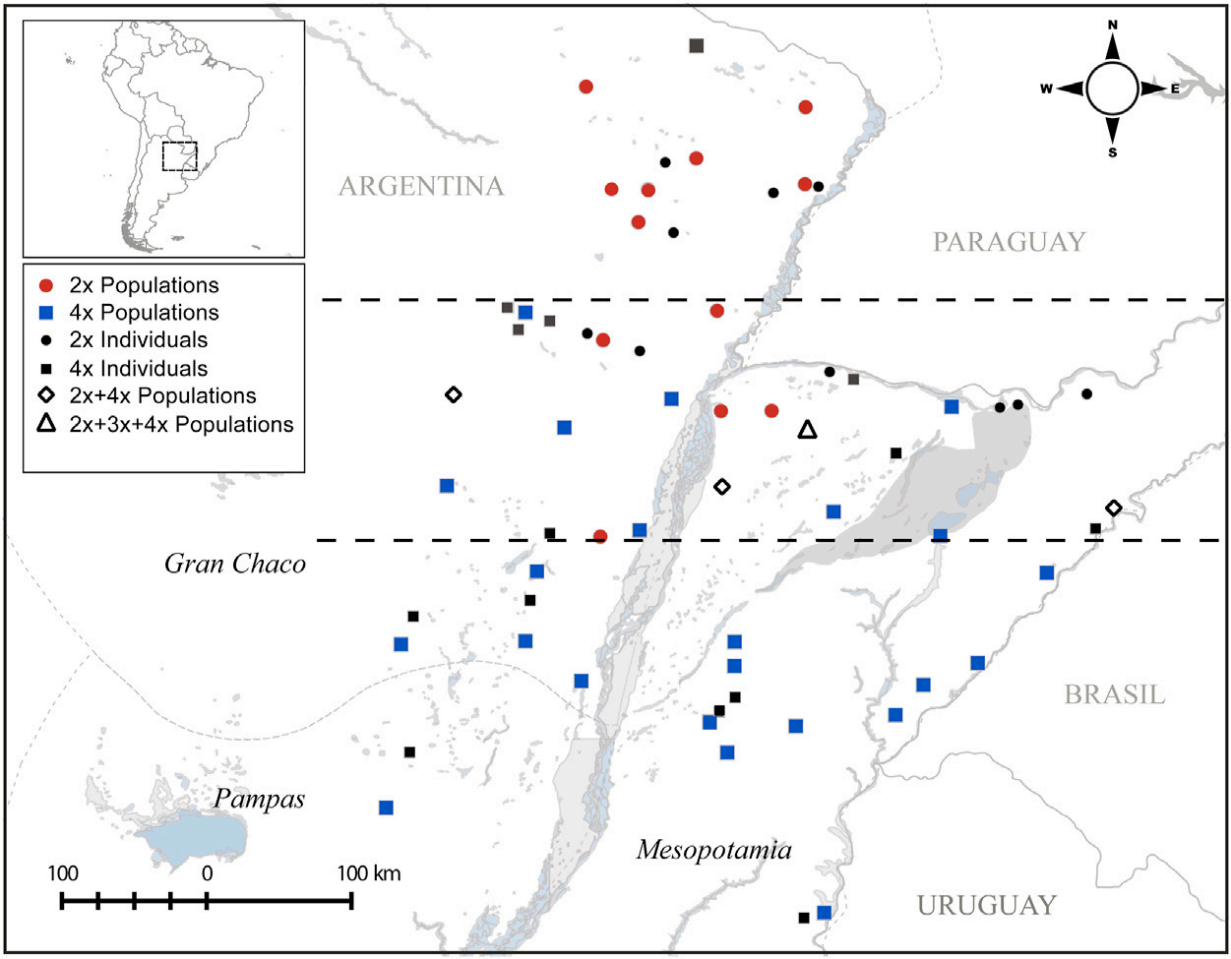
Collection locations of studied *P. intermedium* populations and their ploidy levels (adopted from Karunarathne et al., 2018). The dashed lines demarcate the contact zone of cytotypes in the middle separating the north and south diploid and tetraploid distribution zones, respectively.

Sampling was done covering the core and peripheral distribution of *P. intermedium* (i.e., Pampas, Mesopotamia, and Gran Chaco of Argentina). Leaf materials were collected in silica gel from 35 populations and between 20 and 30 individuals from each population. Flow cytometry ploidy evaluations data are from our previous analyses published in Karunarathne et al. (2018). A total of 915 individuals classified as diploids (2n = 2x = 20) or tetraploids (2n = 4x = 40) and representing 24 pure tetraploid populations, nine pure diploid populations, and four mix ploidy populations were used here to analyze the population genetic structure (see Figure 1 and Table 1).

**TABLE 1:** Populations of *P. intermedium* with different ploidy levels, assessed in the present study for genetic variation. Nei’s gene diversity, genotype diversity, and the number of effective genotypes were calculated as in Nei’s (1987) with amplified fragment length polymorphism (AFLP) markers. The cytotype gene diversity was calculated for each population and averaged to diploid, tetraploid, and mixed-ploidy populations.

Population	Ploidy	No. of individuals^a^	Genotype diversity	No. of effective genotypes (%)	Cytotype gene diversity	Nei’s gene diversity	Fst
Hojs420/1P	2x	26	1.000	26.0 (100%)	0.169	0.165	0.66
Hojs422/1Q	2x	26	1.000	26.0 (100%)		0.208	0.64
Hojs423/1R	2x	24	1.000	24.0 (100%)		0.176	0.7
Hojs425/1T	2x	17	1.000	17.0 (100%)		0.174	0.75
Hojs429/1U	2x	22	1.000	22.0 (100%)		0.157	0.76
Hojs432/1V	2x	26	0.997	24.1 (92.7%)		0.188	0.65
M31/1W	2x	21	1.000	21.0 (100%)		0.173	0.71
M26/1X	2x	11	1.000	11.0 (100%)		0.146	0.78
Hojs468/2S	2x	25	1.000	25.0 (100%)		0.135	0.74
Hojs401/1A^b^	4x	23	0.249	1.3 (5.7%)	0.032	0.098	0.9
M29/1B	4x	26	0.772	3.9 (15%)		0.067	0.79
Hojs402/1C	4x	26	0.000	1.0 (3.8%)		0.000	0.91
Hojs403/1D	4x	30	0.480	1.9 (6.3%)		0.024	0.9
Hojs404/1E	4x	20	0.195	1.2 (6%)		0.026	0.92
Hojs405/1F	4x	26	0.895	7.1 (27.3%)		0.075	0.72
Hojs406/1G	4x	11	0.000	1.0 (9.1%)		0.000	0.9
Hojs409/1H	4x	23	0.000	1.0 (4.3%)		0.000	0.91
Hojs410/1I	4x	28	0.958	13.0 (46.4%)		0.107	0.68
Hojs414/2J^b^	4x	27	0.000	1.0 (3.7%)		0.000	0.91
Hojs415/1K^b^	4x	27	0.359	1.5 (5.6%)		0.034	0.89
Hojs416/1M	4x	24	0.000	1.0 (4.2%)		0.000	0.93
Hojs424/1S	4x	25	0.000	1.0 (4%)		0.000	0.93
Hojs440/2C^b^	4x	24	0.228	1.3 (5.4%)		0.015	0.91
Hojs443/2F	4x	29	0.567	2.2 (7.6%)		0.023	0.9
Hojs445/2H	4x	29	0.488	1.8 (6.2%)		0.039	0.89
Hojs451/2M	4x	28	0.198	1.2 (4.2%)		0.034	0.88
Hojs453/2Ñ	4x	11	0.182	1.1 (10.0%)		0.015	0.86
Hojs455/2P	4x	19	0.105	1.1 (5.8%)		0.054	0.91
Hojs465/2R^b^	4x	17	0.485	1.8 (10.6%)		0.024	0.9
Hojs475/2U	4x	27	0.501	1.9 (7%)		0.024	0.91
Hojs471/2X^b^	4x	19	0.731	3.3 (17.4%)		0.043	0.9
Hojs470/2T^b^	2x,3x,4x	25 (19)	0.810	4.5 (18%)	0.123	0.134	0.79
Hojs456/2Q^b^	2x,4x	25 (20)	0.367	1.5 (6%)		0.137	0.84
Hojs481/2W^b^	2x,4x	23 (5)	1.000	23.0 (100%)		0.147	0.66
Hojs487/2Y^b^	2x,4x	29 (26)	0.820	4.8 (16.6%)		0.075	0.82

a In brackets are the number of polyploids from the total number of individuals.

b Populations located in the contact zone of cytotypes.

### gDNA Extraction and Amplified Fragment Length Polymorphism

The genome DNA of all the collected samples were extracted using QIAGEN mini plant DNA extraction kit (QIAGEN GmbH, Hilden, Germany), from 50 mg of silica-dried leaf materials. The DNA concentrations were quantified and used for AFLP analysis.

For AFLP, we followed the methodology described by Vos et al. (1995) with a modification skipping the pre-selective amplification of the digested fragments. Further, selective primer combinations with four additional bases (instead of three) in one of the primers were used, though not in all combinations (see Supplementary note one for primer details). The three primer combinations used were EcoR I- ACA-5′ FAM/Mse I- GAAC, EcoR I - AATG/Mse I -AAC-5′ HEX, and EcoR I - AGA/Mse I -ACA-5’ TAMRA. This modification yielded consistent DNA fragments, reducing the laboratory time substantially. The reproducibility of PCRs was checked with 10 duplicate samples with each primer pair.

For restriction digestion and ligation, approximately 500 ng of genome DNA of each sample was digested overnight with *EcoR I* (5 Units) and *Mse I* (1 Unit) (New England Biolabs, Frankfurt, Germany), and T4 DNA ligase (Promega Corporation, Mannheim, Germany) (1 Unit) with EcoR adapter pair (5 pmol) and Mse adapter pair (50 pmol) with the presence of NaCl (0.05 M) and BSA (0.05 mg/ml) in 1X ligase buffer (Promega Corporation, Mannheim, Germany). The direct selective amplification reaction mixture consisted of 1X PCR buffer (10x NH_4_ reaction buffer; Bioline GmbH, Luckenwalde, Germany), 2.5 mM MgCl_2_ (Bioline GmbH, Luckenwalde, Germany), 0.2 mM dNTPs (Promega Corporation, Mannheim, Germany), 4 pmol of each EcoR and Mse primers, 1 Unit Taq polymerase (BioTaq–Bioline GmbH, Luckenwalde, Germany), and ca. 80 ng of digested DNA in 25 μl final volume. The PCR was done in a Thermal Cycler (BioRad T100; Bio-Rad Laboratories GmbH, Munich, Germany) with the following program. Denatured at 94°C, 2 min, 9 × (94°C, 1 s; 65°C, 30 s, 1°C/cycle; 72°C, 2 min), 23 × (94°C, 1 s; 56°C, 30 s; 72°C, 2 min), 60°C, 30 min. Amplified fragments were analyzed in an ABI 3130xl Genetic Analyzer (Applied Biosystems Inc. Foster City, CA, United States) with the 500 ROX size standard (Applied Biosystems Inc. Foster City, CA, United States). A total of 887 individual fingerprints were retained after the initial analysis of all the individuals (48 atypical fingerprints were removed). Genotyping and binary presence–absence matrices were assembled in GeneMarker 2.6.0 (Softgenetics, State College, PA, United States), with a threshold of 75 RFU for scoring bands of size range of 100–510 bp (small fragments between 50 and 100 were not considered due to the possibility of non-homologous fragments; Vekemans et al., 2002). All the peaks were checked in the panel editor eliminating non-reproducible bands by comparing against replicated samples. Reproducibility of the data was checked using the error rate with 30 duplicate samples, where the similarity of the scoring (i.e., presence–absence of fragments) was cross-checked. The error rate was less than 0.1%, indicating the high reliability of the data.

### Population Genetic Analyses

The combined binary matrix of the three primer combinations was analyzed with the R-script AFLPDAT (Ehrich, 2007) to calculate diversity indices (i.e., Nei’s gene diversity for each population) and genotype diversity (using Nei’s formula for haplotype diversity; Masatoshi Nei, 1987). The binary matrix was assembled into an individual genotype data object with the R package ADEGENET (which includes a method that can handle clonal data and allows for analyses of mixed-ploidy data sets with a correction for allele copy-number ambiguity in polyploids) (Jombart, 2008), which was used in the rest of the genetic analyses in the R environment (R Core Team, 2016). A Neighbor-Joining tree was constructed using the Prevosti’s Distance Coefficient (a measurement over all the loci of the proportion of unshared alleles) with a bootstrap analysis of 1,000 sample size. The R package POPPR 2.7.1 (Kamvar et al., 2014) was used for the distance matrix and the bootstrapping. Principal coordinate analysis (PCoA) was performed based on pairwise Euclidian distance used in the DAPC (Discriminant Analysis of Principal Component) function of the ADEGENET R package. Analysis of Molecular Variance (AMOVA) was calculated on the discrete dissimilarity matrix with 1,000 permutations. For AMOVA, both ploidy and populations were used as different strata to calculate between ploidy and within- and among-population molecular variance.

**FIGURE 2:**
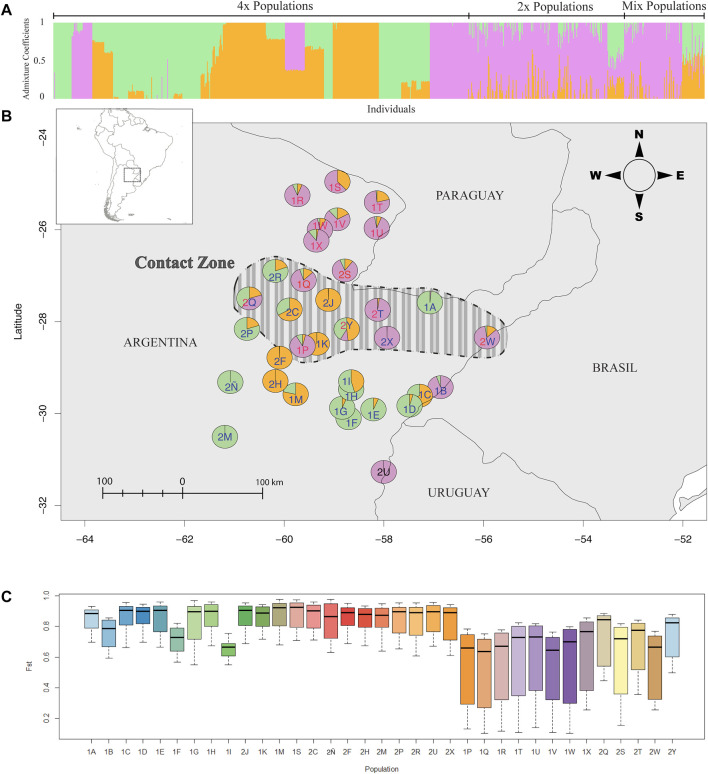
Genetic clusters in *P. intermedium* inferred from AFLPs. **(A)** Bayesian clustering of all the individuals at K = 3. Vertical bars represent the individuals with the proportion of the admixture (i.e., admixture coefficients) in different colors. **(B)** Admixture coefficients of populations plotted on the map indicating the collection location. Pie chart colors follow the different clusters as in **(A)** The color of the population labels indicates the ploidy level of each population (red: diploids, blue: tetraploid, red and blue: mixed populations). The dashed-line area on the map shows the putative zone of contact between parapatric and sympatric diploids and tetraploids. **(C)** Boxplots of the *Fst* values of each population showing their genetic divergence (color scheme only represents populations, and the population order follows the same as 4A).

Bayesian model-based clustering implemented in the “find.clusters” function of R package ADEGENET was used to determine the potential number of clusters that can describe the data best. Here, BICs (Bayesian Information Criteria) are calculated using k-means algorithm (also Ripley’s K-function, where the sum of squares from points to the assigned cluster centers is minimized; Baddeley and Turner, 2005), and the resulting BIC values are plotted against the increasing number of k (clusters). Ideally, the number of clusters where the BIC value starts to increase is taken as the best cluster solution. In our case, the BIC value did not increase (Supplementary Figure S1A). Therefore, according to the plot, any number of clusters more than two and less than 15 will describe the data. Considering the number of groups observed in the NJ tree, k = 3 was taken as the number of clusters, thus making it a biologically meaningful number of clusters. An assignment of three clusters (k = 3) in the STRUCTURE analysis (Figure 2A) best explains the variation. The assignment of *k* = 2 did not noticeably change the admixture values in tetraploids. Clusters were unstable with higher *k* (>3) indicating admixture in almost all individuals. This was also tested with *ad hoc* statistic ΔK based on the rate of change in the log probability of data between successive K values described by Evanno et al. (2005) (Figure S1B). Plotting the genetic clusters was performed using the R package LEA (Frichot and Francois, 2014).

### Isolation by Distance and Environment

A Mantel test was performed using pairwise Euclidean distance (R package VEGAN; Oksanen et al., 2016) to calculate the geographical isolation of each cytotype and population, based on genetic data. The R package MPMCORRELOGRAM (Matesanz et al., 2011) was used to visualize the geographic isolation based on distance intervals. The R package LEA (Frichot and Francois, 2014) was used to plot the genetic admixture on the map.

We also performed environmental association analysis of genetic diversity among populations using bioclimatic data. Bioclimatic data were downloaded from www.chelsa-climate.org (Karger et al., 2017), and point data were extracted for each population. A regression analysis of Nei’s genetic diversity and each bioclimatic variable indicated eight variables showing significant correlation (*p* < 0.05 for all). A forward selection with 95% collinearity was used to sequentially remove intercorrelated variables, and only four variables were retained (Bio1: annual mean temperature, Bio3: isothermality, Bio6: min temperature of the coldest month, Bio15: precipitation seasonality). An environmental distance matrix was obtained using these variables, and a regression analysis of the environmental distance and the genetic diversity of populations was performed to detect isolation by environment.

### Morphological Analysis

During plant material collections, at least three plants were collected from all the studied populations for morphological assessment, and the collected vouchers were deposited in several herbaria in Argentina (MNES, CTES, BAA, and SI) and Germany (B and GOET). Overall, 42 morphological characters (18 quantitative, 24 descriptive/categorical: Table S1) of both diploids and tetraploids were studied from the voucher specimens to discern any morphological variation among cytotypes and populations. A principal coordinate analysis (PCoA) was performed to identify morphological clusters among diploid and tetraploid populations. Similarity percentage analysis (SIMPER) was performed using the software PAST 3.23 (Hammer et al., 2001) to assess the amount of contribution of each morphological character to the observed morphological variation in *P. intermedium* individuals.

## Results

### Genetic Diversity in Cytotypes Across Populations

In the combined binary matrix, a total of 189 fragments were scored; 84 were from the EcoR-Mse (ACA- GAAC), 58 from EcoR-Mse (AATG- AAC), and 44 from EcoR-Mse (AGA- ACA). Out of this, 66.2% (61.6% in 2x, 70.8% in 4x) of fragments were polymorphic. The number of bands per individual ranged from 84 to 91. Cytotype-specific fragments were overall significantly higher in tetraploids (*n* = 41) than in diploids (*n* = 34) (*p* = 0.02).

Diversity analysis (Nei’s gene diversity) showed that all diploids have significantly higher values (paired *t*-test *p*-value < 0.001) in both genotype and gene diversity (Table 1). The effective number of genotypes was maximum in diploid populations (100%), indicating that all individuals were genetically distinct, whereas in tetraploids, the number of effective genotypes varied from 3.7 to 100% (averaging 8.05%; Table 1). Interestingly, mixed-ploidy populations showed variable levels of genotype diversity (between 6 and 100%, averaging 35.2%) which was significantly correlated to the number of diploid individuals in the population (Table 1; Pearson correlations *r* ^2^ = 0.91, *p* < 0.01). One such population (Hojs481/2W: 24-diploids, 6-tetraploids; Table 1) harbored five non-clonal tetraploid individuals, suggesting either independent polyploidization events or (more likely) formation of recombinant polyploids by sexuality. The highest number of effective genotypes observed among tetraploid populations was 13 (46.4%), while six populations showed high *Fst* values (≥0.9) and a single effective genotype (Nei’s diversity = 0.0) and hence were classified as pure clonal populations (Table 1). Grouping populations by cytotype and geographic occurrence within the zone of contact between cytotypes (Figure 1) revealed that tetraploid individuals and populations in the contact zone were genetically more diverse, both in genotype and gene diversity as well as in the effective number of genotypes compared to those out of the contact zone (Table 1, 2).

**TABLE 2:** Genetic variation divided into cytotypes and contact zone.

Population	N (n_ind_)	G	G_eff_	Gene div	Fst
Diploids	9 (22)	1.0	21.79	0.169	0.71
Tetraploids	26 (23.9)				
In the contact zone	10 (23.9)	0.5^a^	4.37	0.071	0.77
Out the contact zone	16 (23.9)	0.33^b^	2.58	0.031	0.82

N: number of populations (n_ind_): average number of individuals per population; G: genotype diversity; G_eff_: number of effective genotypes; Gene div.: mean value of Nei´s gene diversity.

a : G = 0.56 without taking clonal populations

b : G = 0.49 without taking clonal populations.

When taking the complete dataset, AMOVA revealed that half of the genetic variation (50.18%) is found within populations, the rest accounting for among-population variation (ϕ = 0.4102, p = 0.001). When ‘cytotype’ was assigned as the preferred hierarchy, within-cytotype genetic variation was 36% (ϕ = 0.572, p = 0.001). Among-population variation increased to 64.8% in tetraploids when cytotypes were analyzed separately, while the values for diploids did not change noticeably (36%).

### Genetic Clusters and Population Structure Variation

**FIGURE 3:**
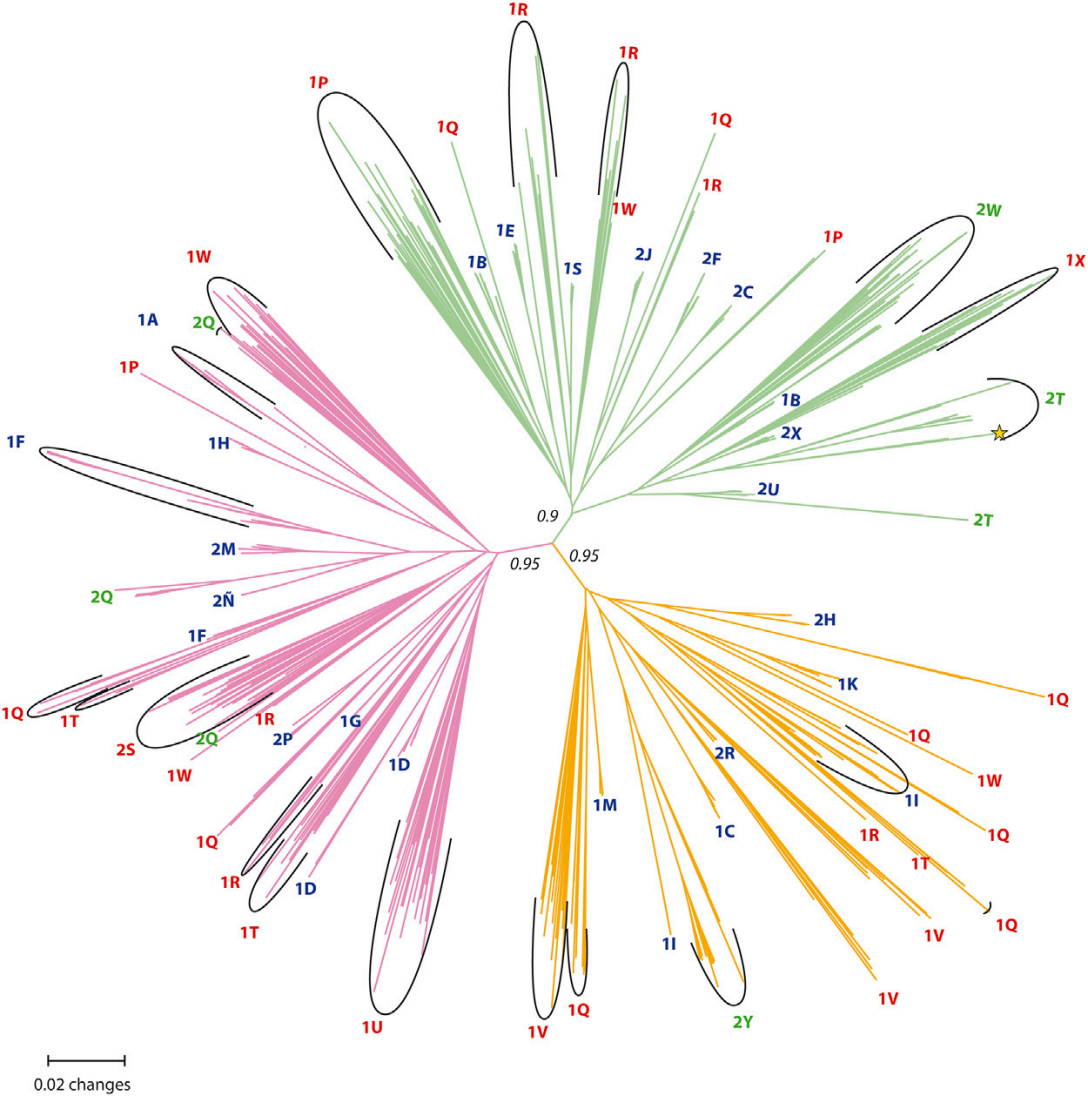
The unrooted Neighbor-Joining (NJ) tree constructed using the Prevosti’s Distance Coefficient among the amplified fragment length polymorphisms (AFLPs) of all the studied individuals of *P. intermedium* with a bootstrap analysis of 1,000 sample size. The bootstrap values are shown only for the main branches. The tip labels show the respective population (red: diploid populations, blue: tetraploid populations, green: mixed-ploidy populations, *: triploid individual).

Three major clusters with strong bootstrap support (>90%) were observed in the unrooted NJ tree (Figure 3), and five (sub-)clusters were resolved with variable bootstrap values (<70%). Pure diploid and tetraploid populations and mixed-ploidy populations were grouped in all three clusters and interspersed among each other (Figure 3), suggesting that tetraploids were formed independently from different diploids within each cluster. In only a few diploid populations, all individuals from the same population were grouped together in a single cluster (e.g., M26/1X, Hojs429/1U, and Hojs468/2S; see Figure 3). In most cases, individuals from the same diploid population formed small groups interspersed among clusters (e.g., Hojs420/1P, Hojs422/1Q, Hojs423/1R, Hojs425/1T, Hojs432/1V, and M31/1W). Individuals from two of such populations were found in two clusters (populations Hojs420/1P and Hojs425/1T), while individuals from three diploid populations were found in three clusters (populations Hojs422/1Q, Hojs423/1R, and M31/1W). Such wide genetic representation observed among individuals of single populations indicates either an ancestral origin or regular gene flow among diploid populations. A Mantel test of genetic variation in diploid populations showed isolation by distance supporting the former hypothesis (*r* ^2^ = 0.163, *p* = 0.031). Contrary to diploid populations, all but three tetraploid populations (populations M29/1B, Hojs405/1F, and Hojs410/1I) formed compact groups within each cluster (Figure 3). Two out of four mixed-ploidy populations (Hojs481/2W and Hojs487/2Y; Figure 3) formed a consistent single group, while individuals from the other two mixed-ploidy populations formed small, separate groups within each cluster (in populations Hojs470/2T and Hojs456/2Q; Figure 3). Mantel test for tetraploids exhibited genetic variation strongly correlated to distance among populations (*r* ^2^ = 0.3040, *p* = 0.001). Furthermore, when the overall distance found among 4x populations and genetic clusters of the studied individuals were plotted against distance intervals (Mantel correlogram; Matesanz et al., 2011), almost all groups (except for two) indicated significant isolation (filled points in Supplementary Figure S2, *p* < 0.05). This is expected in apomictic species where random mating—even within populations—is limited as apomixis imposes substantial reproductive isolation among individuals (see Discussion for details). Our IBE analysis showed that the observed genetic diversity among all populations shows a strong correlation to the environmental distance showing substantial isolation by environment (F-statistic: 6.605, adjust R-squared: 0.1415, *p*-value: 0.0148, Supplementary Figure S4). This observation corroborates with our previous claim of the ecological divergence of cytotypes.

**FIGURE 4:**
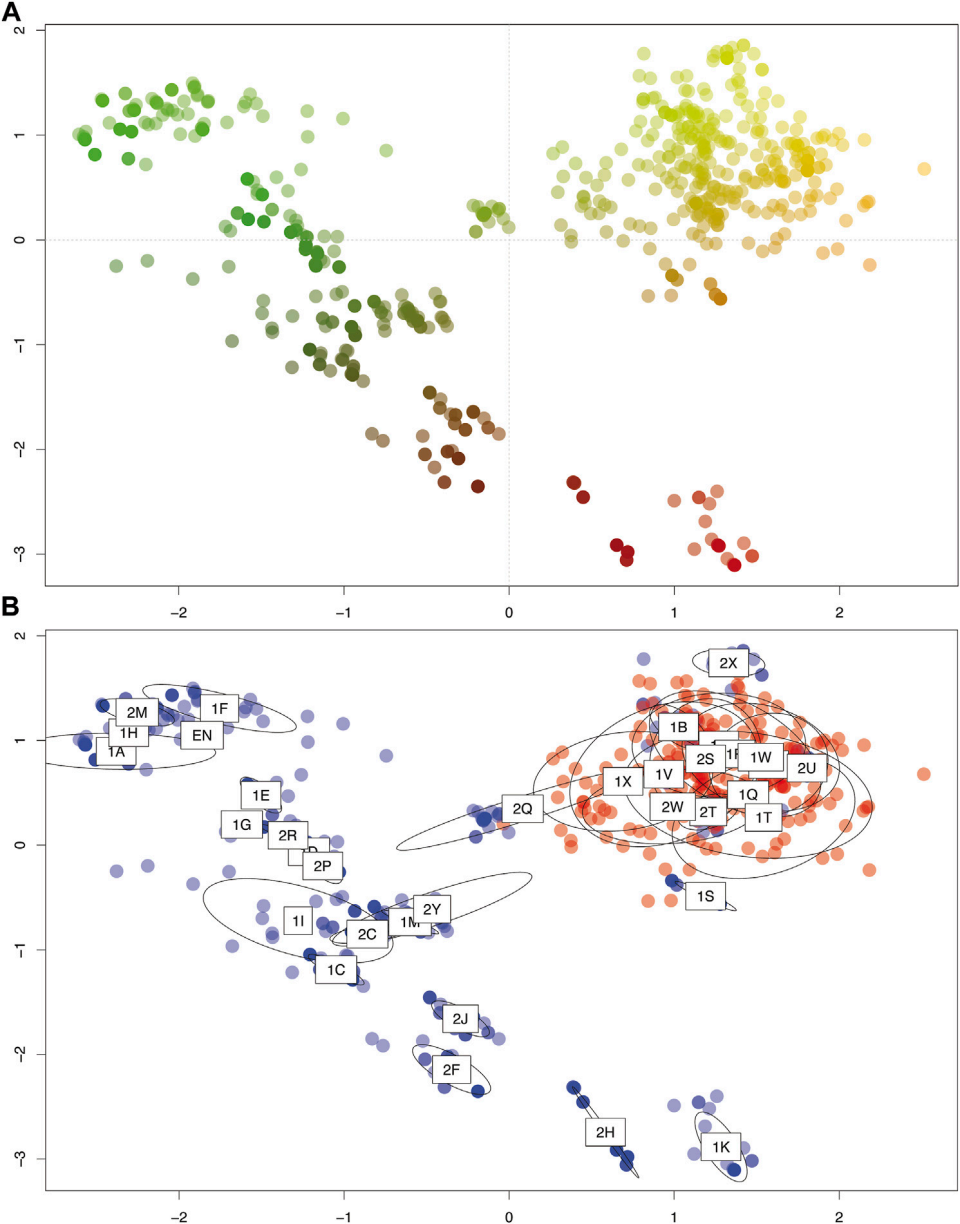
Principle coordinate analysis (PCoA) of the studied populations of *P. intermedium* based on pairwise Euclidian distance used in the DAPC (Discriminant Analysis of Principal Component) function of the ADEGENET R package. The first two axes represent 37 and 21% of total variation, respectively. **(A)** Genetic similarity among all the individuals depicted by RGY color scheme (i.e., Red, Green, and Yellow distinct groups, and the shades of each color show how much related they are to each pure RGY group). **(B)** Genetic variation between cytotypes (red: diploids, blue: tetraploids) and populations (labels indicating population codes are mapped on the center of ellipses representing the 95% dispersion of individuals from each population).

The PCoA revealed higher genetic similarity among diploid populations compared to tetraploid ones (Figure 4). Despite highly clonal populations, tetraploids revealed a wider dispersion along both PCoA axes and a continuous genetic variation (Figure 4). Interestingly, the tetraploid population located in the north of the distribution (Hojs424/1S; see Figure 1) as well as the mixed-ploidy populations Hojs470/2T and the Hojs481/2W clustered together with diploids. The third mixed-ploidy population (Hojs456/2Q) clustered in between the diploids and most tetraploids, while the fourth mixed population (Hojs487/2Y) fell in the middle of the genetic dispersion of tetraploids (Figure 4).

Bayesian clustering revealed three major clusters (*k* = 3) wherein most pure diploid and tetraploid populations were segregated each in individual clusters, while a few pure populations were clustered together with all mixed-ploidy populations in a third cluster (Figure 2A). Cluster analysis also showed high levels of admixture among diploid and mixed-ploidy populations (Figure 2A), including several tetraploid populations close to the contact zone (Figures 2A,B). Supporting the PCoA, the two mixed-ploidy populations (Hojs456/2Q and Hojs487/2Y) falling out of the diploid clustering (Figure 4, and Figure 2B) harbored the greatest amounts of admixture compared to all the other populations. Noticeably, geographically distant tetraploid populations like Hojs451/2M, Hojs453/2Ñ (EN), and Hojs475/2U (Figure 2B) showed minimum or no admixture. The *Fst* genetic divergence values of each population revealed substantially high levels of genetic divergence among most tetraploid populations compared to diploid ones, except for populations M29/1B, Hojs405/1F, and Hojs410/1I whose values were high but more similar to those from diploids (*Fst* = 0.79, *Fst* = 0.72, and *Fst* = 0.68, respectively) (Figure 2C).

### Morphological Variation

All 42 morphological characters were measured in 64 individuals, representing three reference diploids and 61 tetraploids. A *K*-means clustering with *K* = 3 (based on the number of clusters observed in the genetic variation) showed that the morphology of individuals makes three clusters with substantial overlap among clusters (Supplementary Figure S3). This observation proved clearer when the variation was plotted on PCoA ordination plot showing a continuum of character variation among individuals with no correspondence to populations, genetic clusters, or geographic distribution. The SIMPER analysis showed that qualitative (descriptive/categorical) characters contributed the most to the observed morphological variation, although quantitative characters were also adding to the variability. Inflorescence and anthecium shape contributed 45.6 and 18.0%, respectively, to the variation observed among all the individuals (Supplementary Table S1). Among the quantitative traits, the height of the plant and number of racemes were also recognized as substantial discriminating characters of the morphological variation (each contributing 3.8%, and 2.8% respectively; Supplementary Table S1). Analyses of populations with respect to their geographic location (i.e., North, Central, and South of the species distribution) did not expose any affiliation of morphological variation to geography or climatic gradients and rather indicated a wide range of gradual character variations (Supplementary Figure S3).

## Discussion

In plants, the data on genetic composition and population structure variation are a much-needed complement to information about biological traits and are essential information for understanding the recent evolutionary history of any species. In multiploid taxa with cytotypes showing alternative reproductive modes (other than sexuality) and restricted gene flow (e.g., clonal propagation and apomixis), such studies take a new dimension for understanding natural variation and the evolutionary fate of cytotypes within polyploid complexes. The present analysis revises local and regional standing genetic variation in *P. intermedium* populations and associated morphological and reproductive traits. In combination with data from species distribution modeling (Karunarathne et al., 2018), the present study sheds light on the evolutionary aspects of *P. intermedium* life history, suggesting that intraspecific variation is segregated within cytotypes and that recurrent formation of tetraploids allows a range expansion of polyploid cytotypes into new habitats, thus creating genetically divergent lineages and promoting ecologically differentiation.

### Cytotype Genetic Divergence Is Explained by Differential Rates of Sexuality

The results show that fully sexual diploids have relatively few private fragments (17.9%), suggesting that regular gene flow occurs between diploid individuals among populations and there is minimal isolation by distance. Despite the noticeable number of cytotype-specific fragments among genotypes (21.6%), a considerable proportion of fragments (33.8%) were shared between diploids and tetraploids. Since tetraploids in *P. intermedium* are autopolyploids (Norrmann et al., 1989), the observation of ca. 34.2% of unique fragments in polyploids of mixed-ploidy populations (expectedly having a relatively recent origin, see Discussion below) indicates that those fragments are a consequence of post-polyploidization mutations, likely involving genomic rearrangements. Similar cases of rapid sequence rearrangements (indels), genomic and regulatory alterations during formation, and stabilization of allopolyploids and autopolyploids have already been reported in *Paspalum* spp (Calderini et al., 2006; Martelotto et al., 2007; Siena et al., 2016) and other plant systems (Akiyama et al., 2005; Conner et al., 2008) and are responsible for evolutionary novelty (e.g., Ainouche et al., 2003; Hegarty and Hiscock, 2005; Paun et al., 2006). The levels of genetic variation observed in mixed-ploidy populations of *P. intermedium* (Table 1) point toward regular gene flow between ploidies, either through *de novo* formation of polyploids (2x → 4x) or through backcrosses (2x × 4x). The finding of only one triploid individual among the four mixed populations (<1% of all individuals) instead of an expectedly higher number of triploids from backcrosses suggests that 1) there is likely an interploidy barrier between cytotypes (either pre- or post-zygotic) and 2) that rare triploids are more likely to arise from diploids through an unreduced gamete, thus providing support to the hypothesis of *de novo* formation of polyploids *via* a triploid bridge, as postulated for other *Paspalum* species (Quarin, 1992) and recently discussed as a pivotal step enhancing polyploid establishment in nature (Hojsgaard, 2018). The fact that the triploid individual is genetically more related to the diploid individuals in that population than to the coexisting tetraploids further supports this hypothesis and matches the above statement, indicating a probably recent origin for the mixed-ploidy populations.

In pure tetraploid populations, the data show low within-population genetic variation in concordance with the observed low genotype diversity (Table 1), a result consistent with the lack or reduced sexuality inherently associated to high proportions of apomictic reproduction in *P. intermedium* (Karunarathne et al., 2018; 2020b). Studies alike have also shown a reduction in the number of effective genotypes in populations of polyploid apomicts, even when the overall level of genetic diversity is maintained (e.g., Hamston et al., 2018). In *P. intermedium*, the most significant contribution to genetic diversity comes from the variation found among populations, holding an average proportion of private fragments (21.6%). Our results—and particularly the neighbor-joining analysis—further indicate that the observed among population variation in genotype diversity is due to independent polyploid founder events, plus the posterior sporadic occurrence of sexuality. Low proportions of sexual reproduction (<16%) had been detected through embryology on ovules and flow cytometry analysis on seeds of individuals from all tetraploid apomictic populations included here (Karunarathne et al., 2020b). Since apomixis can impose a within-population reproductive isolation among individuals, the rate of (residual) sexuality in such populations has a higher impact on gene flow analysis than geographic distance. The regular occurrence of low rates of sexuality is central to the adaptation and evolution of apomictic populations in agamic complexes (Hojsgaard and Hörandl, 2019). We previously showed that *P. intermedium* tetraploids can have residual reproduction within populations as high as 20% at the seed stage and up to 40% at the embryo-sac stage depending on the environmental stress on the plant (Karunarathne et al., 2020b). Varying proportions of residual sexuality and facultative apomixis have been often reported in other species from genera like *Boechera* spp. (Carman et al., 2019), *Hieracium* spp. (Hand et al., 2015), *Hypericum* spp. (Barcaccia et al., 2006), *Paspalum* spp. (Daurelio et al., 2004), *Potentilla* spp. (Nyléhn et al., 2003), *Pilosella* spp. (Krahulcová et al., 2014), *Ranunculus* spp. (Cosendai and Hörandl, 2010; Cosendai et al., 2013; Pellino et al., 2013), and *Taraxacum* spp. (Vijverberg et al., 2004; Majeský et al., 2012), among others. Strict clonality has been rarely found in nature, obligate apomictic triploids of *Taraxacum officinale* being perhaps one of the few examples (Van der Hulst et al., 2000). The genetic evidence in *P. intermedium* supports this view and points to residual sexuality playing a critical role in maintaining moderate levels of genotypic diversity despite restricted genetic diversity in apomictic populations. Such genotypic diversity supports the range expansions of polyploid genotypes with greater ecological tolerance into new geographic areas (Karunarathne et al., 2018). Since clonal tetraploid populations are distinct to each other and each population exhibits either single or multiple genotypes rather one common genotype and occupies regions differing ecologically, the data endorse the Frozen Niche Variation model (instead of a General-Purpose Genotype model) of genotype dispersal and habitat occupation for the apomictic populations of *P. intermedium*. The fact that genetic variation is significantly associated to environmental differences further supports this inference. The observed positive correlation between Nei’s population level genetic diversity to environmental distance suggests that genetically different clones/cytotypes are being selected to occupy environmentally different areas.

### Genetic Admixture Shows Multiple Polyploid Lineages of Independent Origins

Panmictic (sexual) populations are expected to hold high levels of genetic admixture facilitated by regular random gene flow between individuals (Suda et al., 2007). The study of the spatial distribution of standing genetic variation in natural populations is relevant to identify the relative levels of genetic admixture (Cruse-Sanders and Hamrick, 2004; Matesanz et al., 2011) and to get insights into the mechanisms responsible for it (Ng et al., 2004). Variations in admixture levels can be attributed to an array of factors, historical or current, which interfere the rates of gene flow between individuals directly (e.g., lack of pollinator specificity, pre- or postzygotic isolation) or indirectly (e.g., spatial isolation, phenology) and can provide useful information on key aspects of species life history, such as dispersal, genetic drift, and selection processes.

In the present study, our results from 35 geographically widespread *P. intermedium* populations point to the occurrence of distinct levels of genetic admixture among populations depending upon the ploidy, the occurrence of multiple cytotypes, and the geographic location of the population (Table 1, 2; Figure 2). In populations of *P. intermedium* holding only a diploid cytotype, the variability observed in the Neighbor-Joining tree displays diploid individuals from each population being widespread within and (in some cases) among the three main genetic clusters of our dataset (Figure 3). Diploid populations like Hojs422/1Q, Hojs423/1R, and M31/1W are good examples and a clear evidence of high levels of genetic admixture in diploids, either through gene flow among individuals and/or migrants between populations. The discriminant analysis provides additional support by showing that all diploids are grouped together, and by the lack of populations isolated by distance (Figure 4). Similar levels of admixture are expected for sexual populations and had already been observed between sexual diploids in other agamic complexes (e.g., Paun et al., 2006; Cosendai et al., 2013).

In populations of *P. intermedium* with only one tetraploid cytotype, the situation is contrastingly different. Tetraploids show low admixture and genetic similarity to different diploid genetic clusters (Figure 2, 3). Unlike diploids, tetraploid individuals from single populations are grouped together, in a few cases interspersed with a few diploids in between (population Hojs410/1I; Figure 3) or diploid–tetraploid individuals from other populations (Hojs405/1F; Figure 3). This is expected in asexual lineages where genotypic diversity is constrained by clonality. Our results on ancestry point to the occurrence of at least 16 independent polyploidization events sharing high genetic similarity with 12 diploid populations (Table 3; Figure 3). In a few cases, it was possible to link the origin of a tetraploid population to one (or two) diploid parental individuals (e.g., in the orange cluster, the population Hojs445/2H likely originated from individuals Hojs422/1Q 16/17; Figure 3). A few diploid populations hold individuals sharing alleles with populations of more than one cluster and are putatively involved in the origin of several tetraploid populations (e.g., diploid population Hojs423/1R likely originated tetraploid populations Hojs404/1E, Hojs406/1G, Hojs424/1S, and Hojs414/2J; Table 3). Multiple origins of polyploidy are commonly observed in different plant species with diverse distribution patterns and life histories and are considered an important evolutionary mechanism for diversification (Fehrer et al., 2005; Lo et al., 2009).

**TABLE 3:** Population origin inferred from Bayesian analysis and Neighbor-Joining tree based on genetic distance.

Genetic cluster	Diploid population	Tetraploid population from	
		Diploid origin	Tetraploid origin
green	1Q/1P	1B (rec)	
	1R	1S 1E (rec)	
	1Q/1R	2J (rec)	
	1P	2C (rec)	2F (rec)
	1X	2X (rec)	
	2W	2W 1B	
	2T	2T 2U (rec)	
orange	1Q	2H (rec)	
	1Q/1W	1K (rec)	
	1Q/1T/1R	1L (rec)	
	1V	2R (rec) 1C (rec)	
	2Y	1L	
	1Q/1V	1M(rec)	
pink	1U	1D (rec)	
	1R/1T	1G	
	1Q	2P (rec)	
	2Q/2S/1W/1R	2Q (rec)	
	1Q/1T	1F (rec)	
	2Q	2Q 2Ñ(rec)	2M(rec)^a^ 1F (rec)^a^
	1P	1H (rec) 1A (rec)	
	1W/2Q	2Q (rec)	

Some populations have individuals sharing genetic similarities to distinct diploid populations.

a These populations share a stem with the mixed-ploidy population 2Q; rec: refers to polyploid populations showing recombinant genotypes.

While sexual diploids in *P. intermedium* show no genetic or cytological (discussed above) evidence of introgression from tetraploids, diploids can inject genetic variability into a tetraploid population through recurrent formation of new polyploids. This is very likely the case in all four mixed-ploidy populations that show a higher number of genotypes compared to pure polyploid populations and intermediate fixation indices (*Fst* values, Table 1; see below). All other populations show little or no genotype variability. Variability in these pure polyploid populations can either be generated through post-polyploidization mutations like genome rearrangements (Birchler, 2012; Mandáková and Lysak, 2018) or by genetic reshuffling through sexual reproduction. As discussed above, most apomicts are facultative and exhibit low levels of functional sexuality, including *P. intermedium* (Karunarathne et al., 2018; 2020b). All but six populations represented by a single clone show evidence of genotype diversification by sexual recombination in local populations, and four tetraploid populations are likely originated from polyploid migrants (populations Hojs405/1F, Hojs416/1M, Hojs443/2F, and Hojs453/2Ñ; Table 1, 3). The tetraploid populations most diverse in terms of genotype variability and number of effective genotypes (M29/1B, Hojs405/1F, and Hojs410/1I; Table 1 and Figure 2) show fixation indices alike those found in diploids populations, suggesting lower genetic differentiation. A genome-wide analysis of variation will provide more details on the demography of these cytotypes and the species. Nevertheless, since apomixis is the dominant reproductive mechanism in these tetraploid genotypes, they capture and fix a fraction of the genetic variability present in diploid parentals, including transgressive variation, which explains the wider distribution of tetraploid populations observed in our discriminant analysis (Figure 4) despite their genetic relatedness to different diploid populations (Figure 3).

Admixture in tetraploid apomictic populations is low, and the feeble indication of gene flow in some populations (e.g., Hojs406/1G, Hojs404/1E, and Hojs403/1D) further supports the evidence of genetic isolation by distance among these populations and significant spatial dissociation (*p* = 0.001) (see Supplementary Figure S2). While strong isolation between distant sexual polyploids can lead to directional selection favoring local adaptations (Parisod et al., 2010), in polyploid apomictic *P. intermedium* individuals, isolation by distance between populations is likely reinforced by restrictions to gene flow imposed by the reproductive mode, which might favor stronger population differentiation at regional scales. Overall, the geographical distribution of genetic clusters identified using Bayesian and discriminant analyses and previous evidence of intra- and inter-population cytotype dynamics indicate that selection is acting at distinct geographic scales to sift genotypes better adapted to harsher environmental conditions in southern areas of the distribution. More specific studies including transplantation experiments and exploitation of next-generation approaches (see e.g., Yang et al., 2016) will help in clarifying these points.

### The Case of the Mixed-Ploidy Populations in the Contact Zone

Contact zones represent geographic areas where two or more conspecific individuals of different ploidies come into contact, and therefore, they are excellent locations representing natural laboratories to study the specifics of biological interactions and evolutionary transitions (Lexer and van Loo, 2006). The most relevant mechanisms to be elucidated in a zone of contact between cytotypes are those enhancing differentiation and reinforcing reproductive isolation. Depending on historical aspects, contact zones are of primary or secondary origin (Petit et al., 1999). In the first case, new polyploids emerge within a parental (often diploid) population, while in the latter, allopatric cytotypes (often diploids and polyploids) come into contact after migration or range expansion of populations. Both primary and secondary contact zones are found in natural populations of many polyploid complexes, like in the *Knautia arvensis* agg. (Kolár et al., 2009), in *Aster amellus* (Castro et al., 2012), or in *Galium valdepilosum* (Kolař et al., 2014). In *P. intermedium*, our current genetic analysis shows that all but one mixed ploidy population form a consistent genetic group with conspecific diploids indicating a primary origin (Hojs470/2T, Hojs481/2W and Hojs487/2Y; Figure 3, Figure 4). Previous evidence of polyploid origin, reproductive biology, and cytotype frequencies (Norrmann et al., 1989; Karunarathne et al., 2018, 2020b), including the occurrence of a triploid plant in population Hojs470/2T, point to the occurrence of autopolyploidization events *via* a triploid bridge and further suggest a primary origin for the contact zones. The fourth mixed ploidy population (Hojs456/2Q) shows a more complex pattern in our Neighbor-Joining tree whereby the diploids exhibit high genetic similarity to pure diploid populations M31/1W, Hojs423/1R, and Hojs468/2S, suggesting gene flow between diploids rather than a possible secondary contact with conspecific tetraploid migrants. While inter-cytotype crosses are commonly observed in secondary contact zones in sexual plants (e.g. Zozomová-Lihová et al., 2015), they are much less frequent among sexual–apomictic polyploid complexes mainly because egg cells in apomictic ovules do not receive pollen, and yet, apomicts’ pollen can exert sizeable reproductive interference on sexuals (Hersh et al., 2016). Studies in natural populations from different *Paspalum* species depict a lack of interploidy hybrids, even when they are easily produced under experimental conditions (e.g., Hojsgaard et al., 2008, 2013; Karunarathne et al., 2020a). The lack of triploids, the occurrence of higher number of effective genotypes, and the levels of admixture among tetraploids from mixed-ploidy populations compared to those in pure populations of *P. intermedium* suggest either that tetraploids are regularly formed *de novo* in these contact zones or that the levels of sexuality in tetraploids are higher and recombinant genotypes are produced at higher frequencies than in pure populations (population Hojs481/2W, for instance, contains five different tetraploid genotypes; Table 1). These observations plus the existence of competition and ecological displacement between diploid and tetraploid cytotypes (Karunarathne et al., 2018) and the fact that five out of six tetraploid clonal populations occur southern from the zone of contact between cytotypes suggest that many tetraploid populations have probably originated at the contact zone and eventually spread to the south of the distribution range. In addition, the southernmost tetraploid populations show close genetic affinities to mixed-ploidy populations in the contact zone (population Hojs475/2U to mixed population Hojs470/2T, Hojs451/2M, Hojs453/2Ñ, and Hojs405/1F to Hojs456/2Q, M29/1B to Hojs481/2W, and population Hojs410/1I to mixed population Hojs487/2Y; Figure 3, and Figure 2), which supports the view of tetraploids’ dispersal to the south.

### A Continuum of Morphological Variation Supports Independent Origins of Polyploid Lineages

The analysis of morphological variation found in 42 quantitative and qualitative characters displays the formation of arbitrary groups with substantial overlap among the apomictic populations of *P. intermedium* but lacking correspondence to either genetic clusters or geographical distribution. The variation rather exhibits a continuum of morphological character distribution among all the populations that is rooted in the variation observed for three representatives of the diploid cytotype (Figure S3). While lack of morphologically discrete cytotype groups can be expected in grass species (e.g., Amirouche and Misset, 2007; Pimentel and Sahuquillo, 2008), or in other families (e.g., in the Rosaceae; Bigl et al., 2019), cases of significant morphological differentiation between cytotypes have been observed in many other plant groups (e.g., Segraves et al., 1999; Kao and Parker, 2010), including one species from the genus *Paspalum* (Quarin and Hanna, 1980). Such morphological variation is mostly due to nucleotypic effects (i.e., effects associated with the DNA content in nuclei or the number of monoploid genomes) or to confound phenomena like hybridity (in allopolyploids) or ecological differentiation (Ramsey and Schemske, 2002). Polyploidization can cause immediate divergence in phenotypic traits important to establishment of neopolyploids without affecting significantly genotypic variation (Oswald and Nuismer, 2011). In *P. intermedium*, we observe rather the opposite, no clear morphological divergence but genetically segregant groups among tetraploid populations. Despite tetraploids showing distinct ecological preferences to diploids and occupying differentiated niches (Karunarathne et al., 2018), the multiple polyploidization events from diverse diploid gene pools detected here create morphologically overlapping groups among tetraploids from distant geographical regions. The occurrence of selfing and apomixis in these polyploid lineages further contribute to keep intrapopulation morphological variability low, as observed in other polyploid apomictic complexes like those of the subfamily Malodieae (Campbell and Dickinson, 1990) or in the *Ranunculus auricomus* aggregate (Hodač et al., 2014). The fact that tetraploid apomicts in *P. intermedium* showing overlapping morphology and evidence of single, independent founder events for each of the apomictic populations studied here (with indication of gene flow among populations as discussed above), provides support to the view that the polyploid complex is evolutionarily young. Alternatively, the paucity in genotypic variation despite the observed rates of residual sexuality might be explained by a high formation/extinction rate of polyploid lineages.

## Conclusions

The present study on genetic variation in 35 natural populations of *P. intermedium* shows that apomictic autotetraploids are of multiple independent origins, indicating recurrent polyploidization and subsequent genotype diversification through facultative apomixis. The substantial among-population genetic variation and genetic similarity analyses further point to polyploidization events from genetically distinct diploid populations. The contact zone existing between diploids and tetraploids is primary in origin where tetraploids are persistently formed from diploids. Such polyploids might become established or outcompeted by coexisting diploid parentals, and yet, because of wider ecological tolerance, some tetraploids manage to disperse southward and establish new polyploid populations, clonal or mostly clonal. Apomictic populations display reduced genotype and genetic variability, strict geographic isolation, and high genetic differentiation which support a recent evolutionary origin for the polyploid complex.

## Data Availability

The original contributions presented in the study are included in the article/Supplementary Material, further inquiries can be directed to the corresponding author.
