# Supplementary material for: Single Independent Autopolyploidization Events From Distinct Diploid Gene Pools and Residual Sexuality Support Range Expansion of Locally Adapted Tetraploid Genotypes in a South American Grass
Source: Front Genet. 2021 Oct 4;12:736088. doi: 10.3389/fgene.2021.736088 (PMC8520906; doi:10.3389/fgene.2021.736088)
Supplement: Supplementary file 1 [file DataSheet1.docx]

Table S1. Output of SIMPER analysis showing the contribution values of each morphological character for the separation of the three clusters observed in the morphology analysis of *P. intermedium*

| Taxon | Average dissimilarity | Contribution % | Cumulative % |
| --- | --- | --- | --- |
| Inflorescence shape | 22.49 | 45.61 | 45.61 |
| Anthecium shape | 8.917 | 18.09 | 63.69 |
| Plant height | 1.883 | 3.819 | 67.51 |
| Leaf length (max) | 1.833 | 3.718 | 71.23 |
| Sheath neck color | 1.781 | 3.612 | 74.84 |
| Leaf length (min) | 1.393 | 2.825 | 77.67 |
| Number of racemes | 1.385 | 2.81 | 80.48 |
| Internode internode length | 0.8423 | 1.708 | 82.18 |
| Inflorescence length (max) | 0.8038 | 1.63 | 83.81 |
| Sheath length (mx) | 0.7361 | 1.493 | 85.31 |
| Sheath length (min) | 0.6605 | 1.34 | 86.65 |
| Internode length (min) | 0.5829 | 1.182 | 87.83 |
| Node hairs | 0.4391 | 0.8906 | 88.72 |
| Spikelet color | 0.4304 | 0.873 | 89.59 |
| Spikelet tip shape | 0.3897 | 0.7904 | 90.38 |
| Glume II hairs | 0.3856 | 0.7821 | 91.16 |
| Spikelet length | 0.3723 | 0.755 | 91.92 |
| Node color | 0.3698 | 0.7501 | 92.67 |
| Lemma texture | 0.3678 | 0.746 | 93.42 |
| Cariopsis color | 0.3669 | 0.7441 | 94.16 |
| Spikelet width | 0.3661 | 0.7426 | 94.9 |
| Palea texture | 0.3648 | 0.74 | 95.64 |
| Cariopsis hair | 0.3597 | 0.7296 | 96.37 |
| Glume II nerves | 0.3597 | 0.7296 | 97.1 |
| Spikelet hair | 0.3597 | 0.7296 | 97.83 |
| Spikelet shape | 0.3597 | 0.7296 | 98.56 |
| Basel raceme length | 0.2044 | 0.4146 | 98.98 |
| Ligule hairs | 0.1019 | 0.2066 | 99.18 |
| Inflorescence width | 0.07299 | 0.148 | 99.33 |
| Sheath neck hairs | 0.06872 | 0.1394 | 99.47 |
| Leaf hairs | 0.06793 | 0.1378 | 99.61 |
| Ligule length | 0.06227 | 0.1263 | 99.73 |
| Apical raceme length | 0.05508 | 0.1117 | 99.85 |
| Ligule color | 0.02432 | 0.04933 | 99.89 |
| Leaf width (max) | 0.01891 | 0.03836 | 99.93 |
| Leaf width (min) | 0.0124 | 0.02515 | 99.96 |
| Plant base | 0.01105 | 0.02241 | 99.98 |
| Raceme hair | 0.009528 | 0.01933 | 100 |
| Leaf margin | 0 | 0 | 100 |
| Ligule hairs | 0 | 0 | 100 |
| Ligule type | 0 | 0 | 100 |
| Sheath keel | 0 | 0 | 100 |


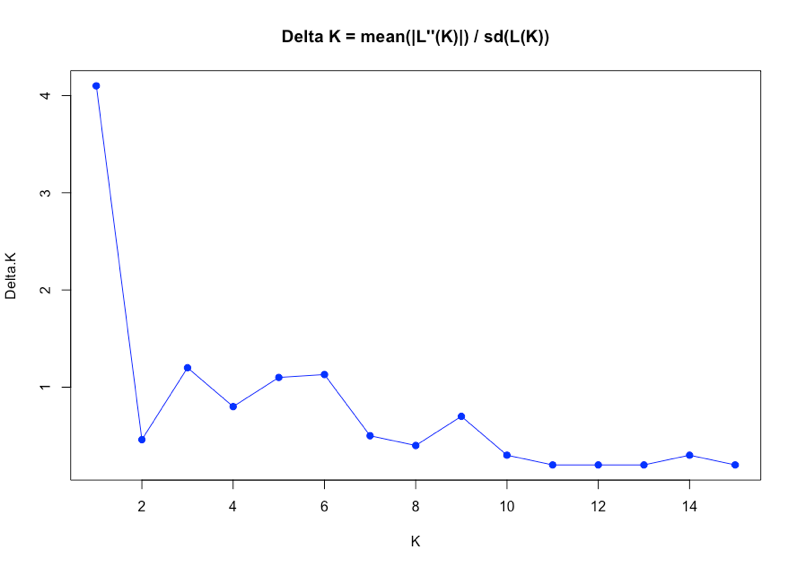


B

A

Figure S1. A. Bayesian model-based determination of potential number of clusters implemented in the “find.clusters” function of R package ADEGENET. A BIC (Bayesian Information Criteria) is calculated using kmeans algorithm (also Ripley's K-function; Baddeley and Turner 2005) and the resulting BIC values are plotted against increasing number of k (clusters). B. Evanno plot (ΔK vs. K) of cluster determination of the AFLP marker data using the method described by Evanno et al. (2005).

Figure S2. Mantel test correlogram showing geographical isolation of *P. intermedium* tetraploids and population based on genetic data; Filled points significant (*p* < 0.05).


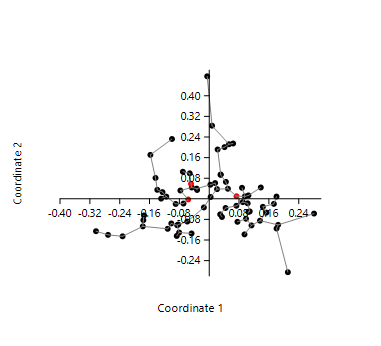

Figure S3. Principle coordinate analysis of the 42 morphological characters of *P. intermedium* individuals assessed in the present study. A. the three-color groups represent arbitrary clusters set by Euclidian optimal clustering (arrow heads show diploids). B. red points – diploids, black points – tetraploids.


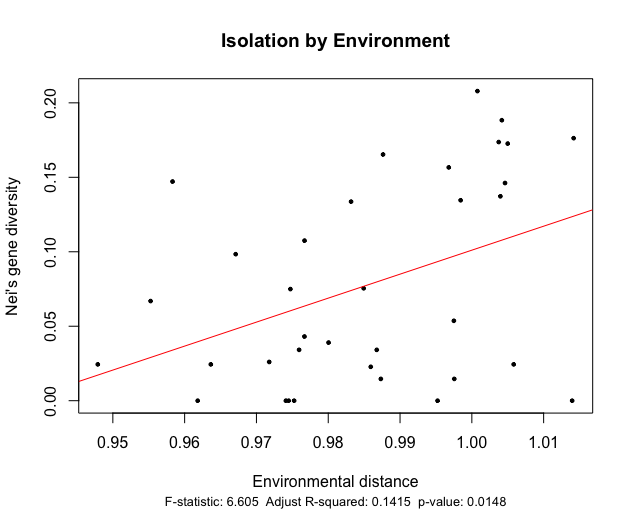


Figure S4. Isolation by environment among the studied populations of *P. intermedium* showing the correlation between genetic variation and the environmental distance of each population.

Supplementary Note 1. Following is a detailed list of AFLP primer combinations used in the study (Fluorescent tag used is indicated at the 5’ end).

Comb. 1

EcoR I gactgcgtaccaattcaaca 5’-FAM

Mse I gatgagtcctgagtaacaac 5’

Comb. 2

EcoR I gactgcgtaccaattcaatg 5’

Mse I gatgagtcctgagtaacaac 5’-HEX

Comb. 3

EcoR I gactgcgtaccaattcaga 5’

Mse I gatgagtcctgagtaacaac 5’-TAMRA
